# Inflammation and dyslipidaemia: a possible interplay between established risk factors in North Indian males with coronary artery disease

**Published:** 2010-04

**Authors:** Binita Goswami, Bhawna Singh, V Mallika, Medha Rajappa, PC Ray, Suresh Kumar

**Affiliations:** Department of Biochemistry, GB Pant Hospital, New Delhi, India; Department of Biochemistry, GB Pant Hospital, New Delhi, India; Department of Biochemistry, GB Pant Hospital, New Delhi, India; Department of Ocular Biochemistry, Dr Rajendra Prasad Centre for Ophthalmic Sciences, All India Institute of Medical Sciences, Ansari Nagar, New Delhi, India; Department of Biochemistry, Maulana Azad Medical College and associated Lok Nayak Hospital, New Delhi, India; Department of Medicine, Maulana Azad Medical College and associated Lok Nayak Hospital, New Delhi, India

**Keywords:** coronary artery disease, C-reactive protein, tumour necrosis factor-α, apolipoprotein-AI, apolipoprotein-B

## Abstract

**Objectives:**

Coronary artery disease (CAD) is a leading cause of morbidity and mortality in the developed world and is rapidly assuming epidemic proportions in developing countries, including India. This has led to extensive research to determine the risk factors and the pathways that may predispose to the elevated risk of this disease. Important among them include lipoproteins, homocysteine, lipoprotein (a), pro-inflammatory cytokines and others. The following study was undertaken to determine a possible inter-relationship between inflammation and dyslipidaemia, which are important risk factors for CAD in the atherosclerosis-prone North Indian male population.

**Methods:**

The study groups comprised 150 clinically assessed North Indian male patients with acute myocardial infarction (AMI), diagnosed on electrocardiographic and biochemical criteria, and 150 healthy controls. Apolipoprotein-AI (Apo-AI), apolipoprotein-B (Apo-B) and C-reactive protein (CRP) levels were estimated using kits based on the immunoturbidimetric assay from Randox, UK. Tumour necrosis factor-α (TNF-α) and lipoprotein (a) were assayed using commercially available ELISA kits from Diaclone Research, Belgium and Innogenetics, Belgium, respectively.

**Results:**

The patients with AMI showed highly significant elevations in the levels of total serum cholesterol, triglycerides, LDL cholesterol, Apo-B and a significant decline in HDL cholesterol, compared with healthy controls. Significantly elevated serum levels of inflammatory markers, TNF-α and CRP were seen in patients with AMI, compared to the control subjects. A significantly positive correlation of TNF-α was observed with lipoprotein (a) in patients with CAD.

**Conclusion:**

The data clearly underlines a possible interplay between inflammation and dyslipidaemia in the pathogenesis of CAD in the Indian context. This insight into the aetiopathogenesis of CAD will prove highly beneficial for devising better preventive measures and pharmacological interventions for CAD.

## Summary

The last century has seen a rapid increase in the global prevalence of coronary artery disease (CAD). Results from the Global Burden of Disease study estimated that India is facing the greatest burden due to CAD.^1^-^3^ It has been projected that by the year 2010, 60% of the world’s patients with heart disease will be in India. Moreover, in India about 50% of CAD-related deaths occur in people younger than 70 years of age, compared with only 22% in the west.^4^ Another interesting feature of CAD among Indians is the lower prevalence of conventional risk factors, such as hypertension, obesity, cigarette smoking, hypercholesterolemia, and others. The explanation for this paradox has been a matter of intense research in the last few decades.^5^

In the recent past, newer risk factors such as lipoprotein (a) [Lp(a)], homocysteine, apolipoproteins, pro-inflammatory mediators, hypercoagulability, platelet aggregation, and insulin resistance have caught the attention of clinicians and researchers as the probable conglomeration of novel risk factors responsible for CAD in Indians.^6^ Hence it is clear that both nature (genetic predisposition) and nurture (environmental or lifestyle factors) are responsible for the disease burden in Asians.^7^

Atherosclerosis, a progressive disease characterised by the accumulation of lipids and fibrous elements in the large arteries, constitutes the single most important contributor to this growing burden of CAD. The hypotheses regarding the pathophysiology of this important malady have evolved substantially over the last few decades. The link between lipids and atherosclerosis has dominated the thinking up to the 1970s, based on strong experimental and clinical relationships between hypercholesterolaemia and atheroma formation. The emerging knowledge on vascular biology led to a focus on growth factors and the proliferation of smooth muscle cells in the 1970s and 1980s. A fusion of these views led to the concept of the atheroma as a graveyard of acellular lipid debris enrobed in a capsule of proliferated smooth muscle cells.^8^ Over the past decade, however, it has been appreciated that inflammation plays a prominent role in atherosclerosis and its complications.

Whereas most clinicians previously regarded atheroma as a bland lesion, the current notion that inflammation and immune response contribute to atherogenesis has garnered increased interest.^9^ A picture emerges of a chronic disease that, from its origin to its ultimate complications, involves inflammatory cells (T cells, monocytes, macrophages), inflammatory proteins (cytokines, chemokines) and inflammatory responses from vascular cells.^10^ Both infection and inflammation induce the systemic host response known as the acute-phase response (APR) and produce many abnormalities that could increase the risk of developing atherosclerosis, including alterations in lipid and lipoprotein metabolism, which is often mediated by cytokines, particularly TNF-α, IL-1 and IL-6.^11^

Hence, atherosclerosis is increasingly being recognised as a complex phenomenon involving the interaction of several mechanisms: dyslipidaemia, inflammation, thrombosis and other dysfunctional metabolic syndromes.^12^ This study attempts to evaluate the contribution of dyslipidaemia and inflammation in the pathogenesis of myocardial infarction and to study a possible interplay between these risk factors in the pathophysiology of CAD.

## Methods

The study population comprised 150 consecutive male first-time acute myocardial infarction patients without past or family history of coronary events, presenting to the medical emergency of Lok Nayak Hospital, New Delhi. Acute myocardial infarction was diagnosed based on clinical, electrocardiographic and biochemical criteria. Patients with a history suggestive of hepatic or renal disease were excluded. Use of lipid-lowering drugs also led to exclusion from the study group.

The patients were enrolled in the study group after giving informed consent and filling in a structured questionnaire, including details of classical risk factors such as family history of CAD, hypertension and smoking. Any patient with a history of diabetes, hypertension, past or family history of CAD were excluded from the study.

The study evaluated the role of inflammation and dyslipidaemia in those patients without apparent risk. The role of smoking could not be excluded from the study groups, as it is very common in India. Body mass index (BMI) values were derived from Quetelet’s formula (weight in kg/height in m^2^). Approval was obtained from the ethical committee of the institution before commencing the study.

Also enrolled were 150 non-diabetic, age-matched healthy controls who satisfied the following criteria: normal glucose tolerance test, absence of angina (Rose questionnaire), absence of history of any vascular disease [acute myocardial infarction (AMI), stroke or intermittent claudication] and normal 12-lead resting electrocardiograms. A patient was diagnosed with AMI if there was a clinical history of ischaemic-type chest pain lasting for more than 20 minutes, substantiated by electrocardiographic evidence of Q waves, ST elevation/depression, and a rise in cardiac troponins/CK-MB.^13^

Arterial hypertension was diagnosed in patients with resting blood pressure values above 140/90 mmHg measured repetitively (at least twice).^14^ Diabetes was diagnosed based on the criteria of the American Diabetes Association expert committee on diagnosis and classification of diabetes mellitus, i.e., fasting plasma glucose ≥ 126 mg/dl, two hours post-load glucose ≥ 200 mg/dl or two random plasma glucose values ≥ 200 mg/dl.^15^

A fasting blood sample was taken and serum was separated and stored at –70°C until the assays were performed. Total cholesterol and triglycerides were measured using commercially available kits on the Olympus AU400 (Hamburg, Germany) auto-analyzer. High-density lipoprotein (HDL) cholesterol was determined, after precipitation of Apo-B-containing particles by phosphotungstic acid-MgCl_2_. Low-density lipoprotein (LDL) cholesterol was calculated for subjects with fasting serum triglyceride levels < 400 mg/dl, using Friedwald’s formula.^16^

Apo-B and Apo-AI were assayed using commercial kits based on an automated immunoturbidimetric method (Randox, UK). C-reactive protein (CRP) was quantitated in the serum using immunoturbidimetric assay kits from Randox, UK. TNF-α levels were measured using commercial ELISA kits from Diaclone Research, Belgium. Lp(a) levels were estimated using enzymelinked immunosorbent assay [Innotest Lp(a), Innogenetics, Belgium]. This method uses mouse monoclonal anti-Lp(a) as solid-phase antibody and sheep anti-Apo-B polyclonal antibody labelled with horseradish peroxidase (HRP) as capturing antibody.

## Statistical analysis

All the values are expressed as mean ± SD. Continuous variables were compared with the Student’s *t*-test. As the parameters followed a non-Gaussian distribution in the study population, Spearman’s rank correlation was used to look for association between different variables in the study group. Univariate logistic regression analysis was performed to ascertain the role of the different risk factors for CAD in our study. A *p*-value < 0.05 was considered significant. Statistical analyses were performed with SPSS for windows version 12 (SPSS Inc).

## Results

The clinical characteristics of the study group are shown in Table 1. Significantly elevated levels of total cholesterol, triglycerides, LDL cholesterol and Apo-B were observed in patients with AMI, compared with healthy control subjects (Table 2). The patients with AMI also exhibited lower HDL cholesterol and Apo-AI, compared with controls (Table 2). However, there was no statistically significant difference between Apo-AI levels of patients and controls.

**Table 1.** Clinical Characteristics Of Study Groups

	*AMI patients (n = 150)*	*Controls (n = 150)*
Age (years)	55.1 ± 9.6	53.7 ± 10.2
BMI (kg/m^2^)	23.2 ± 4.2	22.5 ± 3.9
Systolic BP (mmHg)	128 ± 9.4	121 ± 10.1
Diastolic BP (mmHg)	78 ± 4.5	75 ± 5.2
Smoking (*n*)	10	6
Alcohol intake (*n*)	6	4
Past history of CAD	0	0
Family history of CAD	0	0

**Table 2.** Biochemical Parameters In The Study Groups

*Parameter*	*Patients (n = 150)*	*Controls (n = 150)*	p*-value*
Total cholesterol (mg/dl)	188.6 ± 40.15	145.5 ± 29.71	< 0.001
Triglycerides (mg/dl)	143.3 ± 64.83	123.2 ± 41.92	< 0.001
HDL (mg/dl)	38.2 ± 6.29	43.2 ± 5.57	< 0.001
LDL (mg/dl)	133.9 ± 32.11	79.7 ± 20.23	< 0.001
Lp(a) (mg/dl)	40.2 ± 6.54	10.5 ± 2.34	< 0.001
Apo-A (mg/dl)	107 ± 19.37	109.1 ± 18.69	NS
Apo-B (mg/dl)	99.6 ± 23.55	76.9 ± 22.62	< 0.001
TNF-α (pg/ml)	86.9 ± 14.76	15.2 ± 4.23	< 0.001
CRP (mg/l)	5.01 ± 1.99	1.20 ± 0.28	< 0.001

NS: not significant.

Serum levels of inflammatory markers such as CRP and TNF-α were measured in the study population and they exhibited a highly significant difference between the patients with AMI and the controls. Spearman’s rank correlation showed a highly significant positive correlation between TNF-α and lipoprotein (a), while a similar correlation was observed between CRP and lipoprotein (a) (Table 3). The positive correlation between TNF-α (a pro-inflammatory cytokine) and lipoprotein (a) levels indicates a probable interrelationship between dyslipidaemia and inflammation in the pathogenesis of CAD.

**Table 3.** Spearman Correlation Of The Inflammatory Markers With The Lipid Parameters In Patients With AMI

*Inflammatory parameter*	*Lipid parameters*	r*-value*	p*-value*
TNF-α v/s	Cholesterol	0.058	NS
	Triglycerides	–0.076	NS
	LDL	0.175	NS
	HDL	–0.127	NS
	Apo-AI	–0.031	NS
	Apo-B	0.018	NS
	Lp(a)	0.698	0.003
CRP v/s	Cholesterol	0.070	NS
	Triglycerides	0.159	NS
	LDL	0.047	NS
	HDL	–0.023	NS
	Apo-AI	0.107	NS
	Apo-B	–0.070	NS
	Lp(a)	0.714	0.001

NS: not significant.

Table 4 depicts the odds ratio of the various risk factors analysed by univariate regression analysis. The odds ratio is the measure of the increase in risk of the disease per unit increase of the parameter. In univariate analysis, Lp(a) among the lipid parameters and CRP among the inflammatory parameters emerged as the strongest risk factors. Lp(a) had an odds ratio of 1.217 (95% confidence interval of 1.159–1.279) and CRP had an odds ratio of 2.996 (95% confidence interval of 2.216–4.049)

**Table 4.** Univariate Regression Analysis Of Risk Factors For CAD

*Parameter*	*Exp (β)*	*95% CI*	p*-value*
Cholesterol	1.032	1.021–1.044	0.000
Triglycerides	1.007	1.002–1.013	0.012
LDL	1.032	1.020–1.043	0.000
HDL	0.899	0.855–0.945	0.000
Apo-AI	0.991	0.977–1.006	0.258
Apo-B	1.040	1.025–1.056	0.000
Lp(a)	1.217	1.159–1.279	0.000
TNF-α	1.108	1.055–1.163	0.000
CRP	2.996	2.216–4.049	0.000

## Discussion

The CAD rate in Asian Indians has been increasing rapidly and has reached alarming levels.^1^-^3^,^5^ It is this CAD-prone North Indian population that constitutes the study population in this research. In the last decade, substantial improvements have occurred in the assessment of cardiovascular risk. A better appreciation of the atherogenic effects of well-known cardiovascular risk factors has been accompanied by understanding the sum of these factors; i.e. the global risk profile provides a better predictive power than any single risk factor. In addition, a number of more recently identified and less well-known factors have received intense investigation over the past few years.^17^-^19^

The current view of atherosclerosis is a chronic inflammatory process, developing in response to some metabolic disorders, infections and environmental processes, which initiates and promotes lesion development to the point of acute thrombotic complications and clinical events.^19^,^20^ Clearly, inflammation begets more inflammation.^21^ Substantial advances in basic and experimental science have illuminated the role of inflammation and the underlying cellular and molecular mechanisms that contribute to atherogenesis.^22^-^24^

Many individuals develop CAD in the absence of abnormalities in the lipoprotein profile. The availability of effective therapies for preventing even a first myocardial infarction renders imperative the need to identify at-risk individuals, for concerted intervention, before problems manifest. Based on the evidence supporting the role of inflammation in the pathogenesis of atherosclerosis, inflammatory markers have garnered substantial interest as markers of atherosclerotic risk and add to the information available from traditional measures such as lipid profiles.^25^,^26^ In the present study, the roles of CRP and TNF-α were evaluated as markers of the underlying inflammatory process in North Indian patients with acute myocardial infarction, and their serum levels were significantly elevated in the CAD-prone North Indian patients with AMI, compared to controls.

One of these markers, CRP, has proven remarkably robust as a marker of cardiovascular risk. Plasma CRP, an acute-phase reactant produced primarily by the liver in response to inflammatory cytokines such as IL-6, prospectively identifies asymptomatic individuals at risk for coronary events.^27^ The pro-atherogenic functions of CRP include induction of production of inflammatory cytokines and chemotaxis of monocytes, increased expression of cell adhesion molecules, down-regulation of endothelial nitric oxide synthase expression and activity, increased uptake of LDL-C by macrophages, and stimulation of tissue factor production by peripheral blood monocytes.^28^-^32^

CRP elevation indicates the systemic nature of progressive atherosclerotic disease, which suggests that patients with enhanced inflammation are generally at high risk for progression of atherosclerotic disease and may exhibit multiple vulnerable lesions. The concept of early identification of vulnerable patients who are susceptible to cardiovascular adverse events seems appealing, and measurement of inflammatory biomarkers may be a potent adjunctive tool for this purpose.^33^

Earlier studies such as the OACIS (Osaka Acute Coronary Insufficiency study) and the Quebec Cardiovascular study have shown that CRP levels were significantly raised in the study group, compared to age- and gender-matched controls.^34^,^35^ The JUPITER trial has firmly established the utility of CRP as a biomarker to identify populations that will benefit from preventive therapy. Robert Glynn, the statistician associated with the project, has conservatively estimated that hsCRP screening followed by high-dose statin therapy over a five-year period can prevent more than 250 000 heart attacks, strokes, revascularisation procedures and premature vascular deaths in the USA alone. CRP has now emerged as a new diagnostic and therapeutic modality for the management of coronary artery disease and stroke.^36^ Our study echoes the same finding.

CRP plays a major role in regulating lipoprotein metabolism. It promotes uptake of native LDL-C. CRP has also been shown to significantly reduce cholesterol efflux from THP-1 (human myelogenous leukaemia cell line) and peripheral blood mononuclear cells to apoA-I or HDL.^37^ CRP also decreases the expression of ATP-binding membrane cassette transporter A1 (ABCA1) and ABCG1.^38^

TNF-α is a pro-inflammatory cytokine produced primarily by activated monocytes/macrophages in response to a variety of stimuli.^39^ Recently, TNF-α has been implicated in the pathogenesis as well as the progression of atherosclerotic plaques in a number of ways.^40^ The possible mechanisms postulated include the enhanced surface expression of ICAM-1, VCAM-1, and E-and P-selections on endothelial cells.^41^ It also leads to increased chemokine and scavenger receptor expression.^42^,^43^ Hirschl *et al.* concluded that the extent of changes in serum TNF-α concentration is significantly related to estimates of infarct extent, obtained scintigraphically.^44^

In a study involving South Indian patients with CAD (acute myocardial infarction, unstable and stable angina), Rajappa *et al.* found that the ratios of pro-/anti-inflammatory cytokines in all the study groups increased significantly when patients with unstable angina were compared to other groups.^45^ Ridker *et al.* concluded that inflammation plays a major role in the acute coronary syndromes and that TNF-α gene and protein expression persisted in the myocytes over time, which suggests a possible long-term role of this cytokine in vascular remodelling.^39^

Wojciech *et al.* conducted a study on the role of inflammation in promotion of left ventricular (LV) diastolic dysfunction. The investigators concluded that plasma levels of TNF-alpha and IL-6 were elevated and there was an association between immuno-inflammatory activation, reflected by plasma levels of cytokines, and LV diastolic dysfunction.^46^ The findings of our study further substantiate the evidence in favour of the pro-atherogenic functions of TNF-α. We demonstrated the superiority of the TNF-α /IL-10 ratio in risk stratification of CAD patients in a previous study.^47^

Lp(a) is a complex of apolipoprotein (a) and LDL-C. Apolipoprotein (a) is an atherothrombogenic moiety that can competitively inhibit plasminogen activity, leading to impaired fibrinolysis.^48^ Lp(a) has also been implicated in enhanced oxidation and foam cell formation. Lp(a) functions as a dual pathogen that is thrombogenic, one through its LDL-like characteristics and the other through its plasminogen-like properties.^49^,^50^ It forms a link between genetics and two major explanations of the pathogenesis of atherosclerosis: the fibrin-deposition theory of Rokitansky and the lipid hypothesis of Virchow.^51^,^52^ Recently, it has been proposed that in settings of enhanced oxidative stress and elevated Lp(a) levels, a pro-inflammatory milie may predominate that contributes to the clinical expression of CAD.^53^,^54^

Lipoprotein (a) has a prothrombic role in the pathogenesis of CAD due to its structural homology with plasminogen and it has been hypothesised that the former interferes with plasminogen activation and creates a thrombogenic milieu. It has a tendency to self-aggregate and hence, a greater capacity to bind to glycosaminoglycans and other structures in the vascular wall. Lp(a) binds avidly to endothelial cells, macrophages, fibroblasts and platelets, as well as to the sub-endothelial matrix; and promotes proliferation of vascular smooth muscle cells and chemotaxis of human monocytes. Due to its unique structural homology with plasminogen, Lp(a) competes with plasminogen for binding to plasminogen receptors, fibrinogen and fibrin. It also induces the production of plasminogen activator inhibitor 1 (the main inhibitor of the fibrinolytic system) and inhibits the secretion of tissue-plasminogen activator by endothelial cells.^55^-^58^ All these effects may be potentiated by concomitant dyslipidaemia.^59^

Anuurad *et al.* found that the presence of inflammation as detected by increased levels of CRP and fibrinogen resulted in increased Lp(a) levels among African-Americans.^60^ It was shown that a combination of high Lp(a) levels with a high level of either CRP or fibrinogen was associated with an increased risk for CAD. These results suggest the possibility of an interaction between Lp(a) and inflammatory markers and, furthermore, that the presence of inflammation modulates the risk-factor properties of Lp(a).^60^

Quantification of apolipoproteins A and B provides a measure of the total number of anti-atherogenic and pro-atherogenic particles in plasma.^61^ The Quebec Heart study demonstrated accelerated CAD in patients with hyperapolipoproteinaemia-B.^62^ Our study also showed a significant rise in the atherogenic apolipoprotein B levels in AMI patients in the atherosclerosis-prone North Indian population, compared with control subjects, and indicates that the measurement of apo-B concentration can more accurately delineate coronary artery disease than LDL cholesterol measurement alone.

Yet another finding of our study was the highly significant positive correlation between TNF-α and lipoprotein (a) in the CAD-prone North Indian patients with acute myocardial infarction. The positive correlation between these two parameters throws some light on the complex interaction between inflammation and dyslipidaemia in the pathogenesis of atherosclerosis and its ultimate clinical manifestation, namely, acute coronary syndrome that includes myocardial infarction. The apo(a) gene contains response elements for inflammatory factors such as IL-6.^63^ Ramharack *et al.* reported that Lp(a) and apo(a) mRNA levels in primary monkey hepatocyte culture were responsive to cytokines.^64^

During inflammation, the lipid changes that are observed are not only quantitative but also qualitative, with changes in the composition of lipoproteins. Therefore, the proportion of triglycerides, phospholipids and cholesterol is increased in VLDL, IDL and LDL particles, whereas the proportion of cholesterol and triglycerides decreases in HDL particles.^65^ TNF-α administration in rodents led to an increase in HMG CoA reductase mRNA levels.^66^ In addition to the changes in LDL-C levels observed, a change in LDL size and susceptibility to oxidation of these particles was also observed.

TNF-α levels in humans correlated negatively with particle peak size.^67^ Infection and inflammation was associated not only with a decrease in HDL cholesterol levels but also with a change in the composition. HDLs that circulated during infection and inflammation were depleted in cholesterol esters but enriched in free cholesterol, triglyceride and sphingolipids.^68^ In addition, HDL-associated Apo-AI1 and paraoxonase, lecithin:cholesterol acyl transferase (LCAT), cholesterol ester transport protein (CETP), hepatic lipase (HL), and phospholipid transfer protein (PLTP) levels decreased but Apo-B and serum amyloid A levels increased.^69^,^70^ Because of these changes in HDL metabolism, it is postulated that the main function of HDL, namely its role in protecting LDL against oxidation and reverse cholesterol transport, may be decreased in APR.

The present study highlights significantly higher levels of inflammatory mediators such as CRP and TNF-α in the North Indian male patients with acute myocardial infarction, compared with control subjects, and a highly significant positive correlation between Lp(a) and TNF-α levels in male patients in the atherosclerosis-prone Indian population, clearly pointing to a role in the interplay of inflammation and dyslipidaemia in the pathogenesis of CAD in the atherosclerosis-prone North Indian population.

The strength of this work lies in the fact that the CAD-prone North Indian population constituted the study population and there was a large sample size involved. However, the present study has a few limitations, which include the lack of follow-up data due to patients’ incompliance and administrative constraints, which could have provided precious information about the role of these mediators as markers for risk stratification and prognosis.

Accumulating data indicate that information gained from the link between inflammation, dyslipidaemia and atherosclerosis can yield predictive and prognostic information of considerable clinical utility. New insights into the interplay between dyslipidaemia and inflammation in atherosclerosis may aid in identifying innovative therapeutic strategies to improve outcomes of individuals at risk for or affected by this scourge growing worldwide. We therefore stand on the threshold of clinical application of the interplay between dyslipidaemia and inflammation in atherosclerosis, which could fundamentally alter the way in which we practice preventive medicine, and prove immeasurably beneficial to the public as well.

## References

[1] Murray CJ, Lopez AD (1997). Global mortality, disability and the contribution of risk factors: Global Burden of Disease study.. Lancet.

[2] Achari V, Thakur AK (2004). Association of major modifiable risk factors among patients with CAD – a retrospective analysis.. J Assoc Physicians India.

[3] Mckeigne PM, Miller GJ, Marmot MG (1989). Coronary artery disease in south Asians overseas: a review.. J Clin Epidemiol.

[4] Gaziano TA, Reddy KS, Paccaud F, Jamison DT, Breman JG, Measham AR (2006). Cardiovascular disease.. Disease Control Priorities in the Developing World..

[5] Enas EA, Senthikumar A (2001). Coronary artery disease in Asian Indians. An update and review.. Internet J Cardiol.

[6] Rajappa M, Sharma A (2005). Biomarkers of cardiac injury: An update.. Angiology.

[7] Braundwald E (1997). Heart Disease.. A Textbook of Cardiovascular Medicine,.

[8] Mallika V, Goswami B, Rajappa M (2007). Atherosclerosis – pathophysiology and role of novel risk factors: a clinico-biochemical perspective.. Angiology.

[9] Libby P, Ridker PM, Maseri A (2002). Inflammation and atherosclerosis.. Circulation.

[10] Plutzky J (2001). Inflammatory pathways in atherosclerosis and acute coronary syndrome.. Am J Cardiol.

[11] Khovindhunkit W, Kim MS, Memon RA (2004). Effects of infection and inflammation on lipid and lipoprotein metabolism: mechanisms and consequences to the host.. J Lipid Res.

[12] Harb TS, Zareba W, Moss AJ, Ridker PM, Rifai N, Marder WJ (2003). Association between inflammatory markers, hemostatic and lipid factors in post infarction patients.. Am J Cardiol.

[13] Alpert JS, Thygesen K, Antman E, Barband JP (2000). Myocardial infarction redefined – a consensus document of the joint European Society of Cardiology/American College of Cardiology committee for the redefinition of myocardial infarction.. J Am Coll Cardiol.

[14] Schillinger M, Exner M, Mlekusch W, Sabeti S, Amighi J, Nikowitsch R (2005). Inflammation and Carotid Artery – Risk for Atherosclerosis study (ICARAS).. Circulation.

[15] (1997). Report of the expert committee on the diagnosis and classification of diabetes mellitus.. Diabetes Care.

[16] Friedewald WT, Levy RI, Fredrickson DS (1972). Estimation of the concentration of low-density lipoprotein cholesterol in plasma without use of the preparative ultracentrifuge.. Clin Chem.

[17] Yusuf S, Ounpuu S, Anand S, Sethi KK (1998). Global burden of cardiovascular disease: A review of evidence.. Coronary Artery Disease in Indians: A Global Perspective..

[18] Enas EA, Yusuf S, Mehta JL (1992). Prevalence of coronary artery disease in Asian Indians.. Am J Cardiol.

[19] Wood D (2001). Established and emerging cardiovascular risk factors.. Am Heart J.

[20] Libby P (2003). Vascular biology of atheroscterosis overview and state of the Art.. Am J Cardiol.

[21] Ross R (1993). The pathogenesis of atherosclerosis: a perspective for the 1990s.. Nature.

[22] Buja LM (1996). Does atherosclerosis have an infectious etiology?. Circulation.

[23] Ross R (1999). Atherosclerosis: an inflammatory disease.. N Engl J Med.

[24] Rader DJ (2000). Inflammatory markers of coronary risk.. N Engl J Med.

[25] Alexander RW (1994). Inflammation and coronary heart disease.. N Engl J Med.

[26] McGovern PG, Pankow JS, Shahar E (1996). Recent trends in acute coronary heart disease mortality, morbidity, medical care and risk factors.. N Engl J Med.

[27] Pepys MB, Hirschfield GM (2003). C-reactive protein: a critical update.. J Clin Invest.

[28] Torzewski M, Rist C, Mortensen RF, Zwaka TP (2000). C-reactive protein in the arterial intima: role of C-reactive protein receptor dependent monocyte recruitment in atherogenesis.. Arterioscler Thromb Vasc Biol.

[29] Fichtscherer S, Rosmbingr G, Walter DH, Breur S (2000). Elevated C-reactive protein and improved endothelial reactivity in patents with coronary artery disease.. Circulation.

[30] Venugopalan SK, Devaraj S, Yuhana I, Shaul P (2002). Demonstration that C-reactive protein decreases eNOS expression and bioactivity in human aortic endothelial cells.. Circulation.

[31] Zwaka TP, Hombach V, Torzewski J (2001). C-reactive protein mediated low density lipoprotein uptake by macrophages: implications for atherosclerosis.. Circulation.

[32] Cermark J, Key N, Bach R, Balla J (1993). C-reactive protein induces human peripheral blood monocytes to synthesize tissue factor.. Blood.

[33] Buffon A, Biasucci LM, Liuzzo G, D’onofrio G, Crea F, Maseri A (2002). Widespread coronary inflammation in unstable angina.. N Engl J Med.

[34] Kinjo K, Sato H, Ohnishi Y, Hishida E (2003). Impact of high sensitivity C-reactive protein on predicting long term mortality of acute myocardial infarction.. Am J Cardiol.

[35] St Pierre AC, Bergeron J, Pirro M, Cantin B (2003). Effect of plasma C-reactive protein levels in modulating the risk of coronary heart disease associated with small dense, low density lipoproteins in men.. Am J Cardiol.

[36] Koenig W (2009). Is hs CRP back on board? Implications from the JUPITER trial.. Clin Chem.

[37] Devraj S, Singh U, Jialal I (2009). The evolving role of C-reactive protein in atherothrombosis.. Clin Chem.

[38] Wang X, Liao D, Bharadwaj U, Li M, Yao Q, Chen C (2008). C-reactive protein inhibits cholesterol efflux from human macrophage derived foam cells.. Arterioscler Thromb Vasc Biol.

[39] Ridker PM, Rifai N, Pfiffer M, Sacks F (2000). Elevation of tumour necrosis factor α and increased risk of recurrent coronary events after myocardial infarction.. Circulation.

[40] Gotsman I, Stabholz A, Planer D, Pugatsch T, Lapidus L, Novikov Y (2008). Serum cytokine tumor necrosis factor-alpha and interleukin-6 associated with the severity of coronary artery disease: indicators of an active inflammatory burden?. Isr Med Assoc J.

[41] Oden M (1993). Tumour necrosis factor α as a myocardial depressant substance.. Int J Cardiol.

[42] Pwdil R, Pidrman V, Krejsek J, Gregor J (1999). Cytokines and adhesion molecules in the course of acute myocardial infarction.. Clin Chem Acta.

[43] Stampfer MJ, Sacks FM, Salvini S, Willett WC (1991). A prospective study of cholesterol, apolipoprotein and the risk of myocardial infarction.. N Engl J Med.

[44] Hirschl MM, Gwechenberger M, Binder T, Binder M (1996). Assessment of myocardial injury by serum tumour necrosis factor alpha measurements in acute myocardial infarction.. Eur Heart J.

[45] Rajappa M, Sen SK, Sharma A (2009). Role of pro-/anti-inflammatory cytokines and their correlation with established risk factors in South Indians with coronary artery disease.. Angiology.

[46] Wojciech K, Roksolana D, Monika PK, Alina O, Walentyna M (2008). Plasma levels of TNF-α, IL-6, and IL-10 and their relationship with left ventricular diastolic function in patients with stable angina pectoris and preserved left ventricular systolic performance.. Coron Artery Dis.

[47] Goswami B, Rajappa M, Mallika V, Shukla DK, Kumar S (2009). TNF-α/IL-10 ratio and C-reactive protein as markers of the inflammatory response in CAD-prone North Indian patients with acute myocardial infarction.. Clin Chim Acta.

[48] Scanu AM (1992). Lipoprotein (a): a genetic risk factor for premature coronary artery disease.. J Am Med Assoc.

[49] Luthra K, Misra A, Srivastava LM (1999). Lipoprotein (a): Biology and role in atherosclerotic vascular disease.. Curr Sci.

[50] Rhoads GG, Dahlen G, Berg K (1986). Lipoprotein (a) as a risk factor for myocardial infarction.. J Am Med Assoc.

[51] Rajappa M, Sridhar MG, Balachander J, Sethuraman KR (2006). Lipoprotein (a) and comprehensive lipid tetrad index as a marker for coronary artery disease in NIDDM patients in South India.. Clin Chim Acta.

[52] Dahlen G (1994). Lipoprotein (a) in cardiovascular disease: review article and viewpoint.. Atherosclerosis.

[53] Singh RB, Ghosh S, Niaz MA (1995). Epidemiologic study of diet and coronary risk factors in relation to central obesity and insulin levels in rural and urban populations of North India.. Int J Cardiol.

[54] Watts GF, Gwjlym RM, Mazurkiewicz J (1995). Independent correlation between plasma lipoprotein (a) and angiographic coronary artery disease in NIDDM.. Diabetes Care.

[55] Stampfer MJ, Krauss RM, Ma J, Blanche PJ, Holl LG, Sacks FM (1996). A study of triglyceride level, low-density lipoprotein particle diameter, and risk of myocardial infarction.. J Am Med Assoc.

[56] St-Pierre AC, Cantin B, Dagenais GR, Mauriege P, Bernard PM, Despres JP (2005). Low-density lipoprotein subfractions and the long-term risk of ischemic heart disease in men: 13-year follow-up data from the Quebec Cardiovascular Study.. Arterioscler Thromb Vasc Biol.

[57] Bjornheden T, Babyi A, Bondjers G, Wiklund O (1996). Accumulation of lipoprotein fractions and subfractions in the arterial wall, determined in an *in vitro* perfusion system.. Atherosclerosis.

[58] Tribble DL, Rizzo M, Chait A, Lewis DM, Blanche PJ, Krauss RM (2001). Enhanced oxidative susceptibility and reduced antioxidant content of metabolic precursors of small, dense low-density lipoproteins.. Am J Med.

[59] Galeano NF, Al-Haideri M, Keyserman F, Rumsey SC, Deckelbaum RJ (1998). Small dense low density lipoprotein has increased affinity for LDL receptor-independent cell surface binding sites: a potential mechanism for increased atherogenicity.. J Lipid Res.

[60] Anuurad E, Rubin J, Chiem A, Tracy RP, Pearson TA, Berglund L (2008). High levels of inflammatory biomarkers are associated with increased allelespecific apolipoprotein(a) levels in African-Americans.. J Clin Endocrin Metabol.

[61] Goswami B, Mallika V, Rajappa M, Kumar S, Shukla DK (2008). Apo-B/Apo-A-I ratio: a better discriminator of coronary artery disease risk than other conventional lipid ratios in Indian patients with acute myocardial infarction.. Acta Cardiol.

[62] Lamarche B, Despres JP, Moorjani S, Cantin B, Dagenais GR, Lupien PJ (1995). Prevalence of dyslipidemic phenotypes in ischemic heart disease (prospective results from the Quebec Cardiovascular study).. Am J Cardiol.

[63] Berglund L, Ramakrishnan R (2004). Lipoprotein (a). An elusive cardiovascular risk factor.. Arterioscler, Thrombosis, Vasc Biol.

[64] Ramharack R, Barkalow D, Spahr MA (1998). Dominant negative effect of TGF-ß1 and TNF- on basal and IL-6-induced lipoprotein (a) and apolipoprotein (a) mRNA expression in primary monkey hepatocyte cultures.. Arterioscler, Thrombosis, Vasc Biol.

[65] Cabana VG, Seigel JN, Sabesin SM (1989). Effect of acute phase response on the concentration and density distribution of plasma lipids and apolipoproteins.. J Lipid Res.

[66] Hardardottir I, Grunfeld C, Feingoid KR (1994). Effects of endotoxin and cytokines on lipid metabolism.. Curr Opin Lipidol.

[67] Skoog T, Dichtl W, Boguist S (2002). Plasma tumour necrosis factor-alpha and early carotid atherosclerosis in healthy middle aged men.. Eur Heart J.

[68] Feingold KR, Memon RA, Moser AH, Grunfeld C (1998). Paraoxonase activity in the serum and hepatic mRNA levels decrease during the acute phase response.. Atherosclerosis.

[69] Feingold KR, Judy K, Shigenaga LG (2008). Infection and inflammation decrease apolipoprotein M expression.. Atherosclerosis.

[70] Ly H, Franconi OL, Fielding CJ (1995). Endotoxin and TNF lead to reduced LCAT activity and decreased hepatic LCAT-mRNA levels in Syrian hamsters.. J Lipid Res.

